# Supplementary material for: Unveiling contextual realities by microscopically entangling a neutron
Source: Nat Commun. 2020 Feb 18;11:930. doi: 10.1038/s41467-020-14741-y (PMC7029020; doi:10.1038/s41467-020-14741-y)
Supplement: Supplementary file 1 — Supplementary Information [file 41467_2020_14741_MOESM1_ESM.pdf]

Supplementary Figure

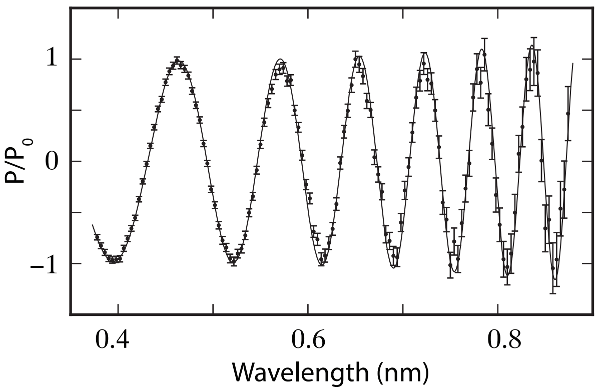

Supplementary Fig 1: **Normalized Neutron Polarization.** A fit of the normalized neutron polarization as a function of neutron wavelength (λ_n) as described in the Methods section. The error bars are standard deviations deduced from counting statistics. For the data shown, the energy phase deduced from the RF frequency was $\gamma = -1.00\pi$. The fitted value for the path phase $is\ \chi = -1.06\pi$ and for the spin plus energy phase is $\alpha + \gamma = -2.01\pi$.
